# Prof. E. R. Peaslee, M. D.

**Published:** 1878-03

**Authors:** 


It is with sincere regret and the most heartfelt sorrow that we record the death of Prof. E. R. PEASLEE, of New York, which occurred at his residence in that city on the 21st of January, of pleuro-pneumonia. Prof. Peaslee was born in Newton, N. H. January 22d, 1814, and had consequently just reached the close of his sixty-fourth year. His academic education was received at Dartmouth College, from which institution he graduated in 1836. In 1840 he received his degree of Doctor in Medicine at the Yale Medical School, and in the following year he commenced the practice of his profession at Hanover, N. H. In 1842 he was made Professor of Anatomy and Physiology at Dartmouth, where he had lectured on these subjects the year previous. This chair he held until 1870. He was made lecturer on Anatomy and Surgery at Bowdoin College in 1843, and held the Professorship of these subjects, there, from 1845 to 1857, from which time until 1870 he occupied the chair of Surgery only.

Prof. Peaslee in 1851 was elected to the chair of Physiology and General Pathology in the New Medical College, and from 1858 to 1860, occupied the chair of Obstetrics at the same institution. The degree of Doctor of Laws was conferred upon him by his *Alma Mater*, Dartmouth in 1859.

It was in the department of Gynæcology that Prof. Peaslee made his most extended reputation. He was made Professor of Gynæcology at Dartmouth in 1872, and at Bellevue in 1874. We believe he also delivered one or two courses of lectures on this subject at the Albany Medical College.

In 1858 Prof. Peaslee moved to New York, where he has since continued to reside. He was held in high estimation by his brethren in the Profession, and was on several occasions elected to positions of honor and trust by them. He was at one time President of the New York Academy of Medicine, was a corresponding fellow of the Obstetrical Society of Berlin, of the Gynæcological Society of Boston, and Honorary Member of the Obstetrical Societies of Philadelphia and Louisville. At the last meeting of the American Gynæcological Society he was elected President.

Prof. Peaslee was a frequent contributor to the various Medical Journals, and his contributions always carried with them the evidence of a ripe experience and a sound judgment. As one of the attending Surgeons at the New York Woman's Hospital, his opportunities for observations were varied and extensive, while his reputation as a discerning diagnostican and a careful operator, gave him a wide field in private practice. In 1857 he published a valuable work on Human Histology, which is now, we believe out of print. In 1872 the crowning work of his life appeared entitled Ovarian Tumors: Their Pathology Diagnosis and Treatment, especially by Ovariotomy. It is not too much to say of this work, that it is the most complete and the best that has been written on the subject.

The loss which the Profession and the world has sustained in the death of Prof. Peaslee cannot be filled. As a physician, as a lecturer and as a man he possessed attributes which are rarely united in one person. His heart was seemingly filled with kindness, and his gentle kindly face reflected it upon all who came in contact with him. The writer of this will long remember the kind, almost fatherly reception which he received at Prof. Peaslee's hands on first entering the Woman's Hospital, in New York. He seemed always ready to do a kind deed or speak a kind word.

In years Professor Peaslee's life was not long, but in deeds it extends far beyond that of many who have lived longer. In the memory of his professional brethren few names will be more tenderly cherished than that of

EDMUND RANDOLPH PEASLEE. B.

